# The Formal Opening of the New Academy of Medicine

**Published:** 1890-12

**Authors:** 


### THE FORMAL OPENING OF THE NEW ACADEMY OF MEDICINE.

The formal opening of the new Academy of Medicine, in New York City, on the evening of the 20th ult., was a most auspicious event, and marks an era in American medicine. The New York Academy of Medicine claims a membership of over seven hundred, including a large proportion of the most eminent of American physicians, and it must be a source of congratulation that it is now at home in its own commodious building. With its great facilities, including its magnificent library of over 50,000 volumes, it must give a new impetus to scientific research, and prove a powerful influence in enlightening the laity and obtaining that recognition, by our lawmakers, which the profession so justly deserves.

RADICAL CURE OF UMBILICAE HERNIA. (*Dr. Annibale Nota. Archivio Ital. di Pediatria, May, 1890.*)

SINCE Desault, at the beginning of the century, proposed the treatment of umbilical hernia in children by external ligature of the sac, many plans have been suggested by surgeons all over the world. Multiple ligature, as practiced by Bonchacone and Martin; transfixion and ligature, according to the plan of Lee; torsion of the sac, instead of transfixion, Thierry's modification; lateral compression of the sac, a suggestion of Chicoinau; the subcutaneous ligature of Holmes, and the cutting operation of

Burwell. These, and others of the same purport, have all proved either inconvenient and objectionable or even dangerous.

Dr. Nota has endeavored to improve the original method of Desault, and has adopted a procedure which he has practiced with complete success in all of the eighteen cases in which he has employed it. The hernial sac, after reduction of the bowel and closure of the ring by the finger, is tightly grasped by an assistant between the thumb and index finger. The base of the sac, as close to the abdominal parietes as possible, is then encircled with three or four turns of an elastic rubber tube of 3 mm. outside diameter. The two ends are tied together and still further secured with a ligature of silk. A loose dressing of cotton is supplied, and the parents are directed to leave the child perfectly free in its movements and without restraint. On the tenth or twelfth day, according to the size of the hernia, the tube is removed, and the sphacelated sac falls off, leaving a small rounded ulcer. This is dressed with iodoform and cotton, and in four or five days is completely healed, leaving a strong, flat cicatrix, which prevents any further protrusion of the intestine at this point.

---

2960. LEUCORRHŒA AND BLENNORRHŒA IN WOMEN.—*Dr. Luaud, in Jour. de Med. de Paris.*

R. Creolin.................................................... gtt. xxx.
Ext. fluid hydr. canad.......................................... fl. drachm ijss.

Sig.—Two teaspoonfuls in a pint of warm water to be used at one injection.

As a urethral injection the following formula is used:

R. Extr. fluid hydrast. canad.................................... gtt. xxx.
Creolin.................................................... gtt. x.
Aquæ.................................................... fl. drachm vii.

Sig.—Use pure as a urethral injection.—*Archives of Gynæcology, etc., Nov.* '90.

### *EXPLANATORY.*

*Owing to the most unfortunate misunderstanding the proceedings of the Tri-State Medical Association were printed in this issue instead of those of the Southern Surgical and Gynecological Associaiton, copy for which was in hands of printers.*

### BACTERIOLOGY.

(123) PEROXIDE OF HYDROGEN AS A DISINFECTANT OF WATER.

*Dr. Altehoefer* (*Centralbl. f. Bakt. und Parasitenk.*, Bd. viii, No. 5, July 25th, 1890, p. 129), after giving references to the literature of the subject, gives his own researches on the disinfective power of $H_2O_2$ dissolved in water. He finds that the addition of 1 per 1,000 to ordinary drinking water, to drinking water containing sewage, or to water containing typhoid bacillus or cholera bacillus, is quite sufficient to destroy the various saprophytic and pathogenic organisms contained under these conditions, if it is obtained perfectly fresh and kept in good condition, and if it is allowed to act for a period of twenty-four hours. It is specially valuable for the disinfection of drinking water because it does not affect the taste, does not alter the color, and in the proportion mentioned is perfectly innocuous. As regards cost, he calculates that sufficient drinking water—say 10 litres—for a family may be sterilized by means of $H_2O_2$ at a cost of about 2d. per diem.—*Supplement to British Medical Journal, Oct.* 25, '90.

ON the evening of the 18th inst., the following were elected officers of the Macon Medical Society:

President—DR. R. O. COTTER.
Vice-President—DR. H. J. WILLIAMS.
Secretary and Treasurer—DR. H. P. DERRY.
Corresponding Secretary—DR. H. MCHATTON.
Reporter—DR. W. A. O'DANIEL.
Librarian—DR. W. F. HOLT.

